# Feasibility study: Use of an optical scanning system to obtain 3D body surface images suitable for total skin electron therapy treatment dosimetry

**DOI:** 10.1002/acm2.70690

**Published:** 2026-07-06

**Authors:** Scott B. Crowe, Naasiha Cassim, Jenna Luscombe, Katie L. McMahon, Tanya Kairn

**Affiliations:** ^1^ Cancer Care Services Royal Brisbane and Women's Hospital Brisbane Australia; ^2^ School of Electrical Engineering and Computer Science University of Queensland Brisbane Australia; ^3^ Queensland University of Technology Brisbane Australia; ^4^ Herston Imaging Research Facility Royal Brisbane and Women's Hospital Brisbane Australia; ^5^ School of Clinical Sciences Queensland University of Technology Brisbane Australia

**Keywords:** Monte Carlo, photogrammetry, total skin electron therapy, TSET

## Abstract

**Background:**

Total skin electron therapy (TSET, also known as TSE, TSI, TSEI or TSEBT) provides an effective treatment for extensive superficial lesions such as mycosis fungoides, although the standing setups and large distances used for treatment mean that comprehensive three‐dimensional (3D) dose calculations are rarely achievable clinically.

**Purpose:**

This study investigated the use of photogrammetry and Monte Carlo simulations as a proposed method to verify TSET treatment dose.

**Methods:**

TrueBeam 6 MeV electron beam phase‐space (PHSP) data supplied by Varian Medical Systems was validated for use in TSET treatment calculations via comparisons with existing TSET commissioning data. The PHSP data was then used to perform Monte Carlo simulations of TSET treatments delivered to a humanoid phantom and eight healthy participants who were all imaged standing in a Vectra WB360 photogrammetry system. Monte Carlo simulation results were visualized for qualitative analysis using in‐house developed Python code, using the matplotlib module and 3D Slicer. The simulation calculations were compared against and past in vivo dose measurements to evaluate the performance of the Monte Carlo simulations in the context of clinical experience of TSET treatment delivery.

**Results:**

The near‐instantaneous acquisition of photogrammetry images made the Vectra WB360 system appealing for potential patient imaging. Monte Carlo simulations generally took less than 2 h per beam and produced 3DDOSE files that ranged in size up to 1.75 GB before being reduced by 99% when summed and exported. Validation calculations generally agreed with measurements in water phantoms and the humanoid phantom, and the participant calculations resulted in a mean B factor of 2.8 ± 0.1 as well as useful illustrations of surface dose homogeneity that showed agreement between participant doses and published in vivo results, in terms of hot and cold spots.

**Conclusions:**

Monte Carlo simulations using the 3D surface imaging data were found to be useful in evaluating surface dose homogeneity and verifying the locations of hot and cold spots, potentially allowing shielding and boost interventions to be pre‐planned. Ideally, 3D patient images derived from photogrammetry (or other surface scans) would be imported into a commercial treatment planning system and used in a more conventional treatment planning and delivery workflow.

## 1 INTRODUCTION

Total skin electron therapy (TSET, also known as TSE, TSI, TSEI or TSEBT) is a radiotherapy treatment modality used in the management of cutaneous T‐cell lymphoma, particularly mycosis fungoides, which can present as extensive rashes, lesions, plaques and superficial tumors covering the skin.^1^, ^2^, ^3^, ^4^ TSET treatments involve the delivery of a whole‐body irradiation of the standing patient using electron beams with large fields and an extended source‐to‐surface distance. The patient stands in various poses during treatment, to enable total skin coverage.^1^, ^2^, ^5^

Unlike contemporary dosimetry techniques that use three‐dimensional (3D) patient images (usually CT scans) and computerized treatment planning systems, TSET treatment times (monitor units, MU) are calculated based on a prescribed dose at a single calibration point on the patient's skin, with reference to phantom measurement data. Two principal issues prevent routine accurate dose calculations for TSET treatments: patient imaging and beam modelling. The patient cannot be CT scanned in their (standing) treatment position, and contemporary treatment planning systems generally do not allow dose calculations for electron beams delivered across large distances or without applicators. Due to these limitations, and the increased potential for set‐up errors during treatment of a standing patient with minimal immobilization, in‐vivo dose measurements are often performed as verification, and to identify areas of under‐dosage that may need dedicated boost treatments or hotspots that may need to be shielded during subsequent TSET fractions.^6^, ^7^ Ideally, the need for manual dose calculations and in vivo verifications would be minimized through the use of 3D calculations of the electron dose delivered to the full extent of each patient's skin surface.^8^, ^9^

Optical surface scanning and photogrammetry technologies create the possibility of producing patient surface models without CT scanning. Synthetic CT or MRI images produced from optically scanned models of the patient provide an alternative for use in dose calculation,^10^, ^11^, ^12^, ^13^ however there are challenges when using handheld cameras or scanners for whole body scans of standing subjects.^11^ Fixed camera photogrammetry systems have been used to capture superficial disease for radiotherapy treatment records^14^ and to acquire images of patients in TSET treatment positions^13^ for projection of Cherenkov converted dose onto patient anatomy.

To date, TSET dose calculation studies have typically simulated dose to phantom or patient data using non‐clinical Monte Carlo simulation software, such as EGSnrc and GAMOS.^8^, ^9^, ^15^, ^16^, ^17^ The modelling of extended SSD electron treatments, potentially including beam degrading screens, is not well supported in commercial treatment planning systems.^16^ For example, in RayStation (v12a), limitations in applicator definition, stand‐off distance between applicator end and dose calculation grid, and the total dose calculation grid volume prevent dose calculation in the conventional TSET geometry.

In addition to simulation of the treatment beam geometry, the optimization of a beam model may require data that is time consuming to obtain. Scanning water tanks are not suitable for the beam arrangements used, as electron beams would be incident on the side of the tank wall, preventing superficial dose measurements. Ionization chamber measurements in water‐equivalent‐plastic slab phantoms and film, thermoluminescent‐ or optically stimulated luminescent‐ dosimeters in or on anthropomorphic phantoms are typically used for TSET beam characterisation.^18^, ^19^, ^20^

The objective of this study was to investigate the use of Varian‐distributed phase space data for Monte Carlo calculations of TSET beams delivered to a rectilinear water phantom, an optically 3D imaged humanoid dosimetry phantom and eight healthy participants, and to thereby demonstrate the feasibility of patient‐specific dose calculations in a geometrically challenging TSET setup.

## 2 METHODS

### 2.1 Simulation imaging

An anthropomorphic RANDO phantom and eight healthy participants were imaged on a Vectra WB360 photogrammetry scanner (Canfield Scientific Inc., Parasippany, USA). The Vectra WB360 whole‐body medical imaging system has previously been used for melanoma detection and longitudinal monitoring of skin lesions.^21^, ^22^ The Vectra system consists of 92 cameras in known, fixed locations surrounding a standing subject. The system uses a photogrammetry technique to reconstruct a 3D model of the subject, including a skin texture. Image acquisition takes 3.5 ms acquisition time and reconstruction approximately 15 min per scan. Reconstructions were generally performed retrospectively, after each participant had left the scanner.

The RANDO phantom was placed on water‐equivalent plastic slabs for surface scanning, to achieve a total height of approximately 165 cm, consistent with setup at time of TSET commissioning measurements performed using the phantom.^20^

All eight study participants were scanned in at least four positions, including the three Stanford positions: anterior and posterior (AP/PA beams), right anterior and left posterior oblique (RAO/LPO beams), and right posterior and left anterior oblique (RPO/LAO beams), mirrored to achieve six positions; and one natural standing “A” pose (with arms by side and feet separated). Participants were asked to wear fitted underwear or fitted activewear, to avoid bunching or draping of clothing as reported by an earlier study.^11^

Reconstructed 3D models of each subject were exported from the VECTRA Analysis Module (VAM) software. Data was exported in STL and OBJ formats, with the latter including an exported JPEG texture map.

### 2.2 Commercial treatment planning system dose calculation

The reconstructed 3D models were imported into RayStation (2025B SP2, RaySearch Laboratories, Sweden) using the in‐built Python scripting API. For each study participant, the VAM‐exported STL model was first repaired using PyMeshFix^23^ to ensure that the imported mesh was comprised of a single triangular mesh. Repaired STLs were imported into newly created “patient” cases with empty CT images, using the following code:

from raystation import *
get_current(“Case”).PatientModel.StructureSets(ct_name).RoiGeometries[roi_name].ImportRoiGeometryFromSTL(FileName = stl_path, UnitInFile = “Millimeter”)

where ct_name was the name of the empty CT dataset, and roi_name was the name of an empty volume. A PMMA spoiler screen volume was manually defined in front of the imported volume. A density and material override of water was applied to the imported volume. The dose for a simple square field with minimal stand‐off was calculated to verify this approach could be used for dose calculations.

Three approaches were sequentially tested to attempt to model the extended SSD TSET delivery: the definition of treatment isocenter at 3 m from center of imported volume, the definition of a 3 m large “external” volume to allow an isocenter with minimal air gap between treatment isocenter and dose calculation volume, and the definition of a custom large nominal field size beam model to the describe spoiler‐scattered treatment beam.

### 2.3 Open‐source Monte Carlo dose calculation

**FIGURE 1:**
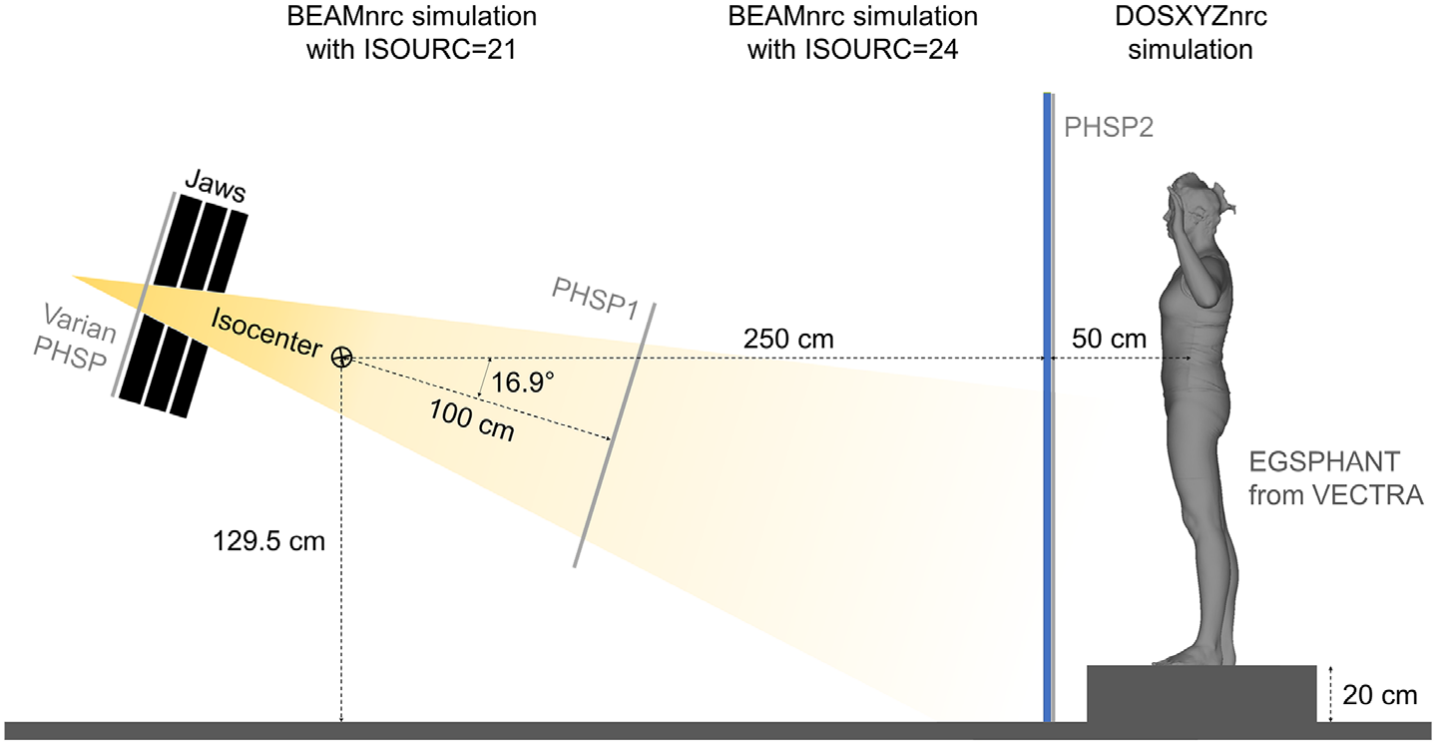
Monte Carlo simulation geometry (note: the floor and platform are included to clarify treatment arrangement, but were not modelled in the simulation geometry).

All Monte Carlo simulations were performed using the EGSnrc software suite, with the BEAMnrc component handling beam production aspects and the DOSXYZnrc user code providing all dose calculations.^24^, ^25^ Phase space (PHSP) data for a 6 MeV beam was taken from the Varian TrueBeam Monte Carlo Data Package (version 2). Dose simulations using these Varian phase space files have previously been validated against measurements.^26^, ^27^ Due to memory constraints affecting the ability to include a spoiler (or six spoiler models, one for each participant orientation) in the simulation models for the phantom and participants, three sets of simulations were performed: two using BEAMnrc and one using DOSXYZnrc, as indicated in Figure 1.

Two BEAMnrc transport geometries were simulated: phase spaces were scored beneath collimators within the treatment head of the linear accelerator, and also beyond a spoiler screen located 2.5 m beyond the linac isocenter, for two angled beams as shown in Figure 1.

The first BEAMnrc simulation modelled collimation of the electron beam in the treatment head. The Varian PHSP file was used as a particle source, using source routine ISOURC = 21. The *Y* jaws, *X* jaws and multi‐leaf collimators were approximated by using the JAWS component module to model three source‐focused paired bars. The separation of these paired bars was 36 cm (projected to isocenter) for the *Y* and *X* jaws, and 42 cm (projected to isocenter) for paired bars approximating the retracted MLCs. A phase space file, PHSP1, was recorded at 100 cm beyond isocenter (146.045 cm from approximated MLC). The space between PHSP1 and JAWS component module was modelled as air, using the SLABS component module.

The second set of BEAMnrc simulations modelled the transport of electrons through the extended SSD geometry and a 6 mm PMMA spoiler screen placed 2.5 m from the linac isocentre. This spoiler screen is used to improve surface dose homogeneity. PHSP1 was used as a particle source, using source routine ISOURC = 24. This source routine allows a rotation around isocentre to be applied, to simulate the two gantry angles used for each dual‐field irradiation with a single phase space file, PHSP2: 16.9° above and below horizontal (e.g., 253.1° and 286.9°).^20^ The point of rotation was defined as the isocentre, 100 cm from the PHSP1 location. The air and PMMA screen were modelled using the SLABS component module.

DOSXYZnrc was first used to simulate the dose for dual‐field irradiations on the surface of a large 200 × 20 × 5 cm^3^ (height, width, depth) Virtual Water phantom located at 50 cm from spoiler screen, replicating a measurement geometry used during commissioning of the TSET technique. Subsequently, DOSYXZnrc was used to simulate dose in a RANDO phantom (with six 60° rotations to simulate anterior, posterior, and oblique positions), and to simulate dose to each participant (using obtained VECTRA images with and without 180° rotations, to simulate anterior and posterior deliveries). No shielding was included in any of these simulation models.

For the RANDO phantom and study participants, the EGSphant dose simulation geometry was produced from the STLs exported from the VAM software. This process was automated using in‐house developed Python code. PyMeshFix^23^ was used to ensure that the imported mesh was comprised of a single closed solid object. A voxel‐based three‐dimensional Boolean mask which defined points enclosed within the imported and repaired mesh was produced using the PyVista library,^28^ using the select_enclosed_points function on a uniform 3 mm resolution grid. This grid was exported as an EGSPHANT file, consisting of air with a density of 0.001 g cm^−3^ outside the masked volume, and water with a density of 1.0 g cm^−3^ inside the masked volume.

Simulations were performed on a desktop PC with an Intel i7‐12700K CPU. BEAMnrc and DOSXYZnrc files were programmatically generated for each EGSPHANT file, and a batch file produced to execute each job. The number of histories simulated in BEAMnrc and DOSXYZnrc were 2 × 10^9^ and 2.5 × 10^8^, respectively.

### 2.4 Dose evaluation

The Python code was used to parse 3DDOSE data and export it using compressed NumPy format (npz) for efficient storage^29^ and to parse EGSLST files to determine simulation times.

Relative dose simulations in the large water phantom were compared against measurements performed at time of commissioning of the TSET technique, at heights of 27.5‐209.5 cm from the floor. Subsequent Monte Carlo results were calibrated consistent with the AAPM Report 23 protocol,^1^ by normalization against the simulated calibration point dose in the large water phantom (at isocenter), resulting in a distribution of values of the B factor, the ratio of surface dose from the planned to treatment beams to dose at a reference point.^1^ The result of summation of these normalized dose values for each of the six dual‐field irradiations for the RANDO phantom or a particular participant, at a given surface location, should result in B factors falling between 2.5 and 3.1.^1^

The six dual‐field B factor distributions in the RANDO phantom were summed, and comparisons made against a percentage depth dose (PDD) profile acquired at time of commissioning of the TSET technique.^20^

Summation of six dual‐field 3‐dimensional B factor distributions was not possible for study participant calculations, as the anterior‐posterior pose and two oblique poses were not co‐registered, due to variations in limb positions between poses. Instead, B factors were manually sampled and summed for points of interest for the participant images for which in‐vivo dose measurements have previously been reported for patient treatments.^7^ This was achieved by converting the B factor distributions into the Nrrd file format using the pynrrd module,^30^ and sampling within 3D Slicer (version 5.4.0) using mark‐ups on the 3‐dimensional visualization of dose.

Sampled calculated B factors at non‐reference anatomical locations were normalized against calculated B factors at the reference location (anterior abdomen), to provide an estimate of dose relative to prescription. These were compared against previously reported measured point doses,^7^ and a Welch's *t*‐test performed to test whether the two datasets had equal means, which would suggest the calculated dose values are consistent with past measurements. Due to the number of comparison sites (forehead, shoulder, hip, etc.) and thus increased chance of a false positive in testing this hypothesis, Šidák corrected significance thresholds of *α* = 0.00005 (***), *α* = 0.005 (**) and *α* = 0.0026 (*) were used. This comparison of simulation calculations and past in vivo measurements enabled the performance of the Monte Carlo simulations to be evaluated in the context of clinical experience of TSET treatment delivery.

## 3 RESULTS

### 3.1 Simulation imaging

**FIGURE 2:**
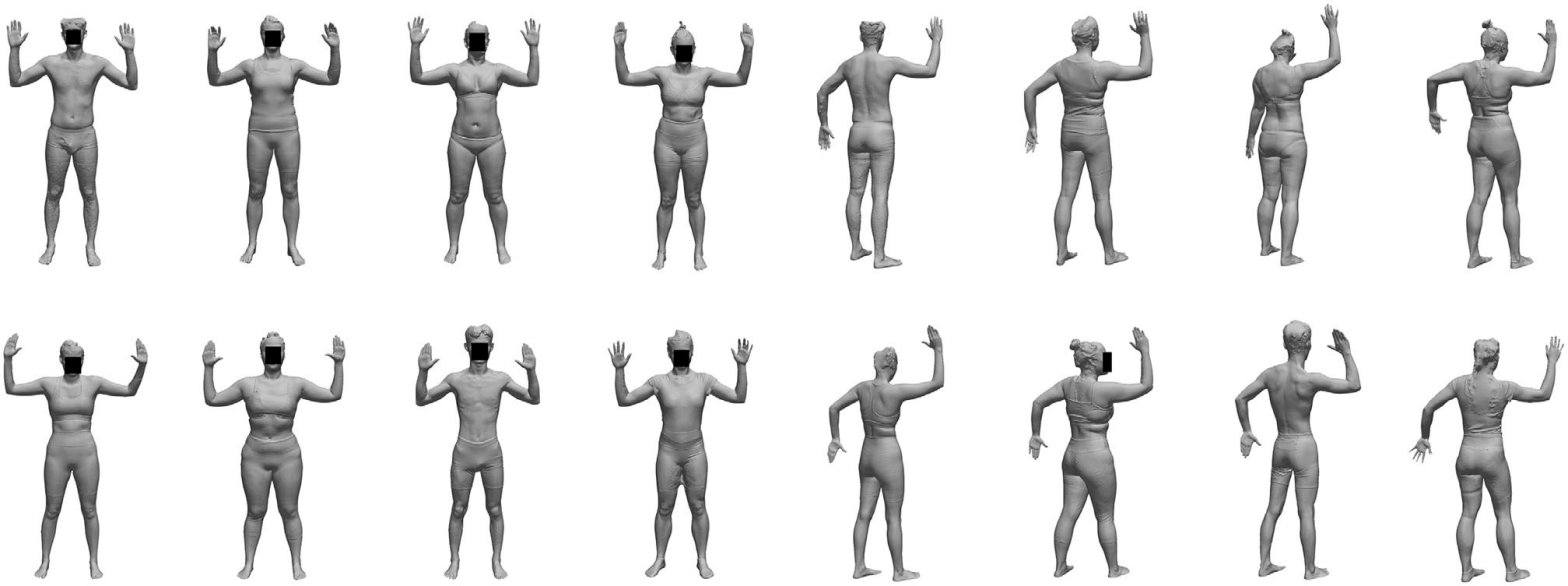
Example anonymized whole body scans of eight participants in anterior and right posterior oblique Stanford poses. Facial features have been hidden to preserve participant anonymity.

Example 3D models of the participants positioned in Stanford poses are shown in Figure 2. Participant 3D surface data file sizes ranged from 10 to 23 MB when exported using the STL format, and from 92 to 155 MB when exported using the OBJ format with JPG textures. As with other optical scanning systems,^11^ hair was often inaccurately reconstructed by the Vectra system.

After each participant was positioned, one set of 92 photographs for photogrammetry took less than a second to acquire, relieving concerns about the need to stand perfectly still for extended periods that hampers the use handheld 3D surface scanning methods for TSET imaging.^11^ One participant, a radiation therapist, reported that they believed patients would have no trouble tolerating the photogrammetry image acquisition, given how quickly the positioning and photography could be performed.

### 3.2 Commercial treatment planning system dose calculation

**FIGURE 3:**
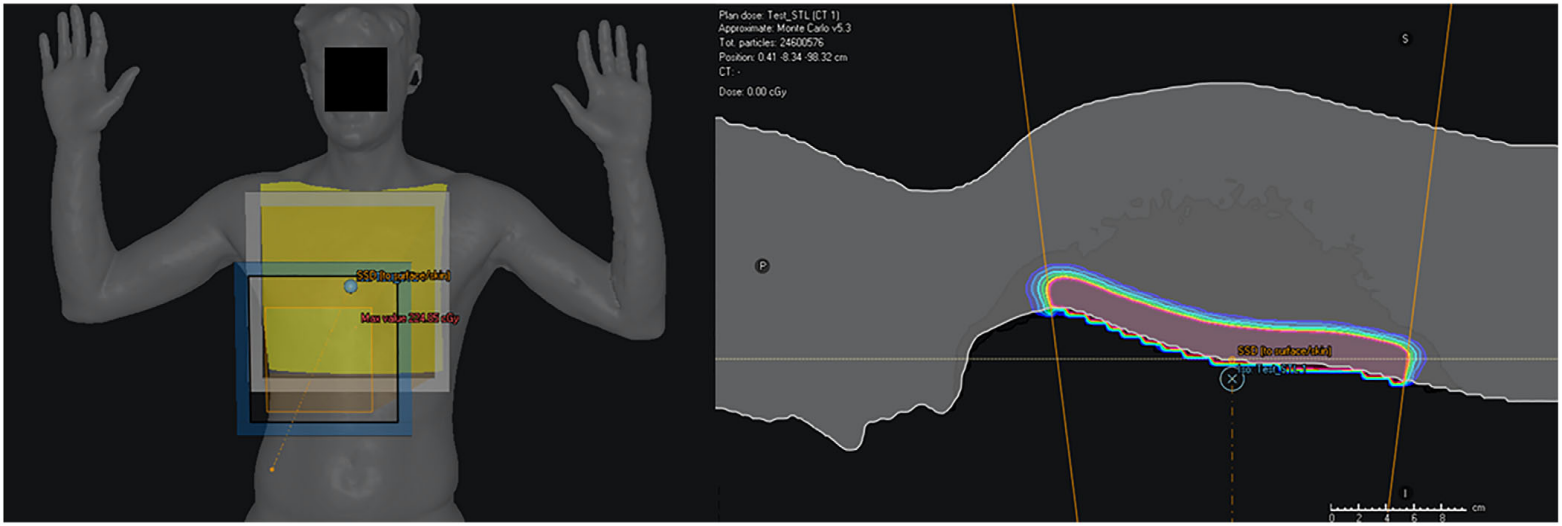
Whole body scan of participant imported into RayStation for electron Monte Carlo dose calculations, with a 6 MeV beam and a 25 × 25 cm^2^ applicator.

An example 6 MeV Monte Carlo dose calculation is shown in Figure 3, illustrating how Vectra‐derived models could be imported and used within the RayStation treatment planning system.

The three attempts to model the extended SSD treatment geometry in RayStation were unsuccessful. The use of an isocentre location 3 m from the imported volume resulted in an “air gap is too large for beam” error. Attempting to reduce this air gap with a large (mostly empty) external volume was not possible due to an upper limit of 2 m on manual volume specification. A customized large‐field beam model was not possible due to limits on scraper/applicator positions.

### 3.3 Open‐source Monte Carlo dose calculation

The simulation of dose using Monte Carlo methods required substantial disk space and processing time. EGSPHANT geometries produced from the participant models ranged from 56 to 177 MB. The average DOSXYZnrc simulation time for each simulated field across the 41 phantom and participant geometries was 1.98 ± 0.98 h, and these simulations resulted in 3DDOSE files ranging from 565 MB to 1.75 GB. Approximately 99% of the space occupied by these simulation results was recovered when dose results were summed and exported as npz files.

### 3.4 Dose evaluation

**FIGURE 4:**
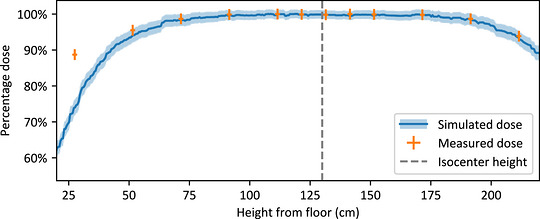
Surface dose profiles in 200 × 20 × 5 cm^3^ virtual water phantom.

The simulated dose to the large water phantom is shown in Figure 4 in comparison with physical ionization chamber measurements from TSET commissioning.^20^ There is a disagreement between Monte Carlo results and physical measurements nearest the floor (i.e., in the inferior region) that is not present at heights above the isocenter.

**FIGURE 5:**
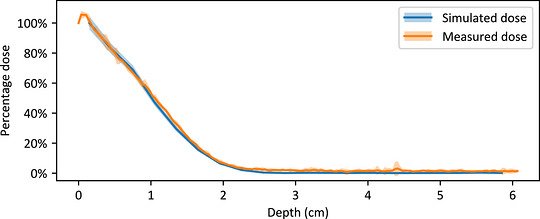
Percentage depth dose in RANDO phantom for six dual field irradiations normalized to 100% at surface.

Figure 5 shows simulated percentage dose profiles at isocenter for a six dual‐field treatment of the RANDO phantom, in comparison to radiochromic film measurement from TSET commissioning.^20^ Note that the film measurement is normalized to 100% at the phantom's surface, resulting in doses greater than 100% in and slightly beyond the very shallow build‐up region of the 6 MeV TSET beam beyond the spoiler. The shallowest point in the Monte Carlo depth dose profile is at 1.5 mm depth, due to the 3 × 3 × 3 mm^2^ voxel size used in the Monte Carlo calculations. The effective R90 and Bremsstrahlung dose at 5 cm for this treatment arrangement were 3.2 mm and 3.3 mm, and 0.6% and 0.1%, for radiochromic film measurements and Monte Carlo simulations respectively.

**FIGURE 6:**
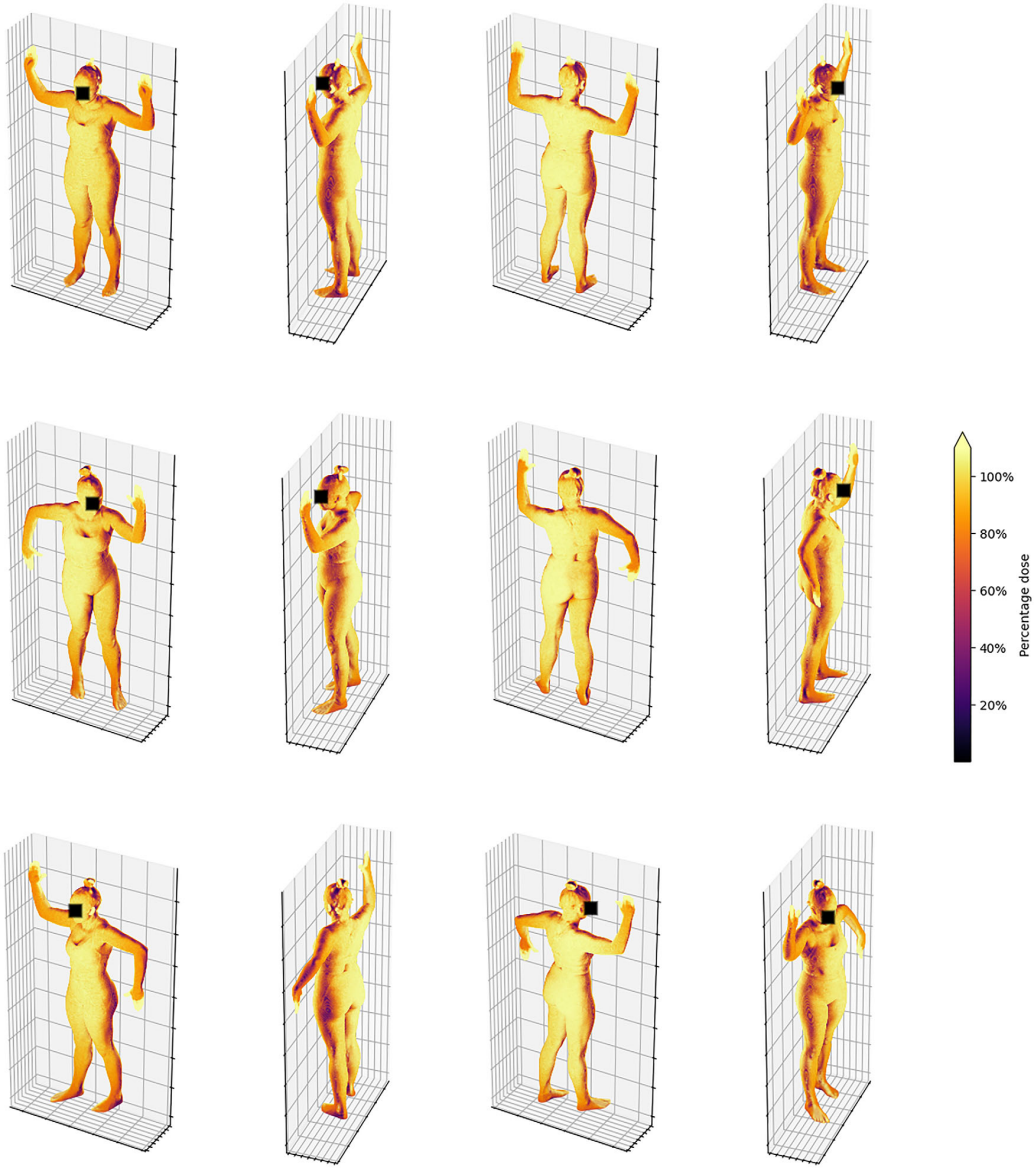
Example paired dual‐field dose distributions for participant 4, in AP/PA position (top), RAO/LPO position (middle) and RPO/LAO position (bottom). Facial features have been hidden to preserve participant anonymity.

Whole‐body surface dose plots from all paired dual‐field irradiations are exemplified for one participant in Figure 6. For each surface dose plot, a normalized dose limit of 110% has been used to increase visible contrast. Higher doses up to 200% were observed in areas of limited thickness that would be irradiated by both the anterior and posterior TSET beams, including the hands, fingers and ears. Surface dose plots for other participants are provided as Supporting Materials 1.

The mean total B factor at the reference point (anterior abdomen), averaged over all participants, was 2.8 ± 0.1. Table 1 shows the result of averaging relative dose calculations (B factor ratios equating to percentages of reference point or nominal prescription dose) across the eight participants of this study, at a range of non‐reference anatomical locations. For comparison, Table 1 also includes results previously measured in vivo during TSET treatments.^7^ Briefly, this previous in vivo dosimetry study involved performing 107 sets of optically stimulated luminescence dosimetry measurements for nine TSET patients treated between 2013 and 2018 using the same TSET technique as simulated here.^7^

**TABLE 1:** Comparison of mean (±1 standard deviation) Monte Carlo calculated dose from the participants in this study against dose results previously measured in vivo during TSET treatments, as a percentage of nominal prescription.

Anatomical region	Calculated dose (%)	In‐vivo measured dose (%)	*p*‐value
Cranial vertex	48 ± 18	72 ± 14	n.s.
Forehead	94 ± 10	94 ± 3	n.s.
Post. neck	99 ± 6	97 ± 24	n.s.
Shoulder	59 ± 11	90 ± 13	***
Hip	87 ± 10	94 ± 7	n.s.
Axilla	72 ± 22	70 ± 28	n.s.
Perineum	36 ± 14	16 ± 7	n.s.
Inner thigh	83 ± 8	42 ± 37	n.s.
Palm of hand	90 ± 9	89 ± 30	n.s.
Back of hand	88 ± 8	79 ± 21	n.s.
Sides of hand	121 ± 16	113 ± 34	n.s.
Elbow	86 ± 6	88 ± 25	n.s.
Inner elbow	86 ± 12	76 ± 20	n.s.
Outer elbow	92 ± 12	114 ± 10	*
Mid. Thigh	96 ± 4	88 ± 12	n.s.
Knee	89 ± 5	103 ± 12	n.s.
Lower leg	84 ± 4	96 ± 10	**
Ankle	79 ± 9	100 ± 14	**
Top of foot	88 ± 6	116 ± 10	***

Data in Table 1 indicate that the Monte Carlo calculated dose at the shoulder, outer elbow, lower leg, ankle and top of foot were not consistent with earlier reported measurements. Dose at cranial vertex, shoulder, axilla, perineum, sides of hand, inner elbow and outer elbow were associated with large standard deviations, indicating variation in dose at these locations across the healthy participants and previous patients.

## 4 DISCUSSION

The importation of surface geometry data from photogrammetry into a commercial treatment planning system has been demonstrated in this study (see Figure 3) confirming that data from the Vectra WB360 system can be used to produce treatment planning system dose calculations that similar to the 3D surface scanner treatment planning demonstrations provided by previous authors.^10^, ^11^ The obstacles encountered when attempting to use the treatment planning system to calculate dose with a TSET beam arrangement (large electron field, no applicator and especially treated model centered 3 m from isocenter), seem to confirm Ding et al.’s 2021 observation, “there is no treatment planning system that can calculate patient‐specific skin dose distribution in TSET”.^16^ Following in Ding et al.’s footsteps, this study used Monte Carlo simulations to achieve 3D dose calculations of TSET treatments when the same result could not be achieved with a commercial treatment planning system.^8^, ^16^

While the Vectra WB360 system has been reported to have a high degree of geometric accuracy^21^, ^22^ an inherent uncertainty was introduced by the need to calculate participant doses in this study with a resolution no finer than 3 mm. This placed the shallowest calculated doses at 1.5 mm depth, somewhat deeper than the basal skin layer. Nonetheless, Monte Carlo simulation results achieved in this study were in broad agreement with previous TSET commissioning measurements and in vivo dosimetry results for patients treated with the setup simulated in this work.^7^, ^20^

The mean B factor at the reference point averaged over all participants in this study, 2.8 ± 0.1, was consistent with the B factor of 2.7 measured using a 30 cm cylindrical phantom during TSET commissioning.^20^

The shape of the PDD in the RANDO phantom derived from combining all simulated TSET beam doses (shown in Figure 5) is qualitatively similar to TSET PDDs reported previously^1^, ^8^, ^18^, ^20^ and the measured Bremsstrahlung dose (shown beyond 3 cm depth in Figure 5) is consistent with previously reported Bremsstrahlung doses^31^ and exceeds the simulated dose in this study. The difference between measured (0.6%) and simulated (0.1%) Bremsstrahlung is within the combined uncertainties in the two values and shows the slight over‐response expected when radiochromic film is calibrated using an electron beam and used to measure Bremsstrahlung photons.^32^, ^33^ Rodrigues et al.^26^ reported agreement between measurements and Bremsstrahlung dose simulations within 2% using Varian phase space data.

The disagreement between measured and simulated dose in the inferior region of the large water phantom (shown in Figure 4) and the disagreement between the participant simulation results and the previous patient measurements in the lower leg, ankle and foot (shown in Table 1) is consistent with reported floor scatter effects.^17^ Agreement could be improved by modifying the EGSnrc simulation geometries, by including the floor and the 20 cm block that patients stand on during TSET treatments. This would enable scatter from these objects to be included in the dose calculations, inevitably increasing the simulated dose to the inferior parts of the water phantom and the participant's lower leg, ankle and foot. Avoiding this modification this instance had the advantage of to allowing the use of a single phase space file (with 180° rotation) to simulate dual‐field irradiation, but was also necessitated by egsphant compilation and DOSXYZnrc calculation memory limits.

With the exception of the lack of scatter in the lower extremities, the Monte Carlo simulation results generated using the Vectra WB360 images of the healthy participants generally agreed with the corresponding published in vivo measurements, confirming the dose hotspots observed at the elbows and hands (see Table 1) and highlighting the elevated dose that would be received by the external ear and fingertips if not shielded (see Figure 6).^7^ The Monte Carlo simulations also indicated dose cold spots at regions identified via in vivo measurements with a similar degree of variation between different patients and participants (see means and standard deviations for the armpit, perineum and cranial vertex in Table 1).^7^

Table 1 shows calculation results at the shoulders and outer elbow that are significantly less than the in vivo measurement results at the same locations. The shoulder and outer elbow are both convex surfaces with comparatively small radii of curvature. In these locations, the optically stimulated luminescence chips used for in vivo dosimetry can stand up a little proud of the skin's surface, leading to a reduction in the beam occlusions that can occur with changes between anterior and posterior treatment positions; the chips can sometimes be irradiated from both sides. The Monte Carlo simulation results being calculated directly at the skin's surface, where occlusion of beams from either one side or the other is more obviously apparent. If calculations of the specific conditions affecting dosimetry measurements is desired, it may be advisable to place dosimeters on participants prior to optical imaging so that this geometry is taken into account in the simulations.

A key limitation of this study arose from the limited vertical field of view provided by the Vectra photogrammetry system. Inability to image anatomy located outside the system's approximately 190 cm vertical field of view meant that it was not possible to simulate a rotational TSET delivery, with each participants hands held above their heads as achieved in Miao et al.’s structured light scanning study.^9^ Rotational TSET deliveries have the advantages of simplifying patient setup and reducing the amount of time that each patient needs to stand, by removing the need to reposition the patient between each beam‐pair delivery, while potentially allowing a more homogeneous skin dose distribution to be achieved.^9^ Without the ability to use the Vectra system to image participants with arms held over their heads, this study instead used established Stanford TSET poses^1^ to mimic the treatment delivery method used locally and in previous TSET commissioning and in vivo dosimetry studies.^7^, ^20^

The inaccessibility of a consistent hands‐above‐head participant position limited the systematic aggregation of limb surface doses simulated across the different Stanford positions, so that comparisons were limited to dose sums at specific points rather than illustrations of the total 3D surface dose. There is potential, however, to use skeletal rigging techniques to facilitate transformation of a model into arbitrary poses. Using this approach, it would be possible to acquire a single scan of a patient, then simulate treatment positions after scanning. This approach could also be used to more easily sum surface doses in three dimensions or correct any errors in positioning during scanning, to achieve more complete and reliable 3D surface dose displays. This approach was presented by Zhu et al.^13^ to align 3D patient models to surface dose maps as captured by Cherenkov cameras.

Ideally, the proposed rigging method would be used to register each participant/patient's internal anatomy obtained via a conventional supine CT scan to their external contour obtained via optical imaging in their TSET treatment position(s). This would enable the effects of underlying density heterogeneities (bone, air cavities) on surface dose to be included in Monte Carlo calculations. The inclusion of internal anatomy in the dose calculation model would also allow the downstream effects of TSET beams to be investigated more fully, potentially encouraging the use of beam energies above 6 MeV to treat cases of more deeply seated mycosis fungoides.^20^

Alternatively, as an extension of the current work, density heterogeneities could be introduced programmatically (e.g., a 0.6 cm layer of bone at 0.6 cm depth in the location of each participant's skull or a 4 cm sphere of bone at 0.5 cm depth at the location of the ankle) or via a more sophisticated machine learning technique such as suggested by Douglass et al.^12^

The absence of shielding from any of the simulation models used in this study was advantageous for providing an up‐front indication of where shielding might be needed if each participant proceeded to TSET treatment, however in future it might be advantageous to include shielding in dose calculation models to help optimize shielding design.

The whole‐body Vectra WB360 imaging system used in this study, or the structured light^9^ or fixed surface imaging systems^13^ evaluated by others, may also be useful for staging and monitoring disease progression and/or treatment response,^14^ by periodically quantifying the degree and extent of surface irregularity or redness affecting the TSET patient.

## 5 CONCLUSIONS

The Vectra WB360 photogrammetry system is capable of achieving comfortable near‐instantaneous acquisition of TSET patient surface geometry data, for potential use in dosimetry and disease monitoring. Monte Carlo simulations using the 3D surface imaging data have been shown to be useful for evaluating surface dose homogeneity and verifying the locations of hot and cold spots, allowing shielding and boost interventions to be pre‐planned. Ideally, the 3D patient images derived from Vectra photogrammetry (or other surface scans) of the standing patient would be imported into a commercial treatment planning system for routine calculation, recording and approval of planned dose from these extended‐SSD large field electron radiotherapy treatments.

## AUTHOR CONTRIBUTIONS

**Scott B. Crowe**: Conceptualization; formal analysis; investigation; methodology; project administration; software; validation; visualization; writing—original draft. **Naasiha Cassim**: Investigation; writing—review and editing. **Jenna Luscombe**: Project administration; writing—review and editing. **Katie L. McMahon**: Resources; validation; writing—review and editing. **Tanya Kairn**: Investigation; methodology; validation; writing—original draft; writing—review and editing.

## ETHICAL APPROVAL

The study was approved by the Royal Brisbane and Women's Hospital Human Research Ethics Committee (LNR/2020/QRBW/66064).

## CONFLICT OF INTEREST STATEMENT

The authors declare no conflicts of interest.

## DATA SHARING

Data that support the findings of this study are available in the supporting material and from the corresponding author, Scott B. Crowe, upon reasonable request.

## FUNDING INFORMATION

The authors received no financial support for the research, authorship, and/or publication of this article. This research was conducted at the Herston Imaging Research Facility utilising infrastructure funded by the Australian Cancer Research Foundation and the Australian Centre of Excellence in Melanoma Imaging and Diagnosis.

## Supporting information



Supporting information: acm270690‐sup‐0001‐SupMat.pdf.
