# Supplementary material for: Feasibility study: Use of an optical scanning system to obtain 3D body surface images suitable for total skin electron therapy treatment dosimetry
Source: J Appl Clin Med Phys. 2026 Jul 6;27(7):e70690. doi: 10.1002/acm2.70690 (PMC13338102; doi:10.1002/acm2.70690)
Supplement: Supplementary file 1 — Supporting information: acm270690‐sup‐0001‐SupMat.pdf. [file ACM2-27-e70690-s001.pdf]

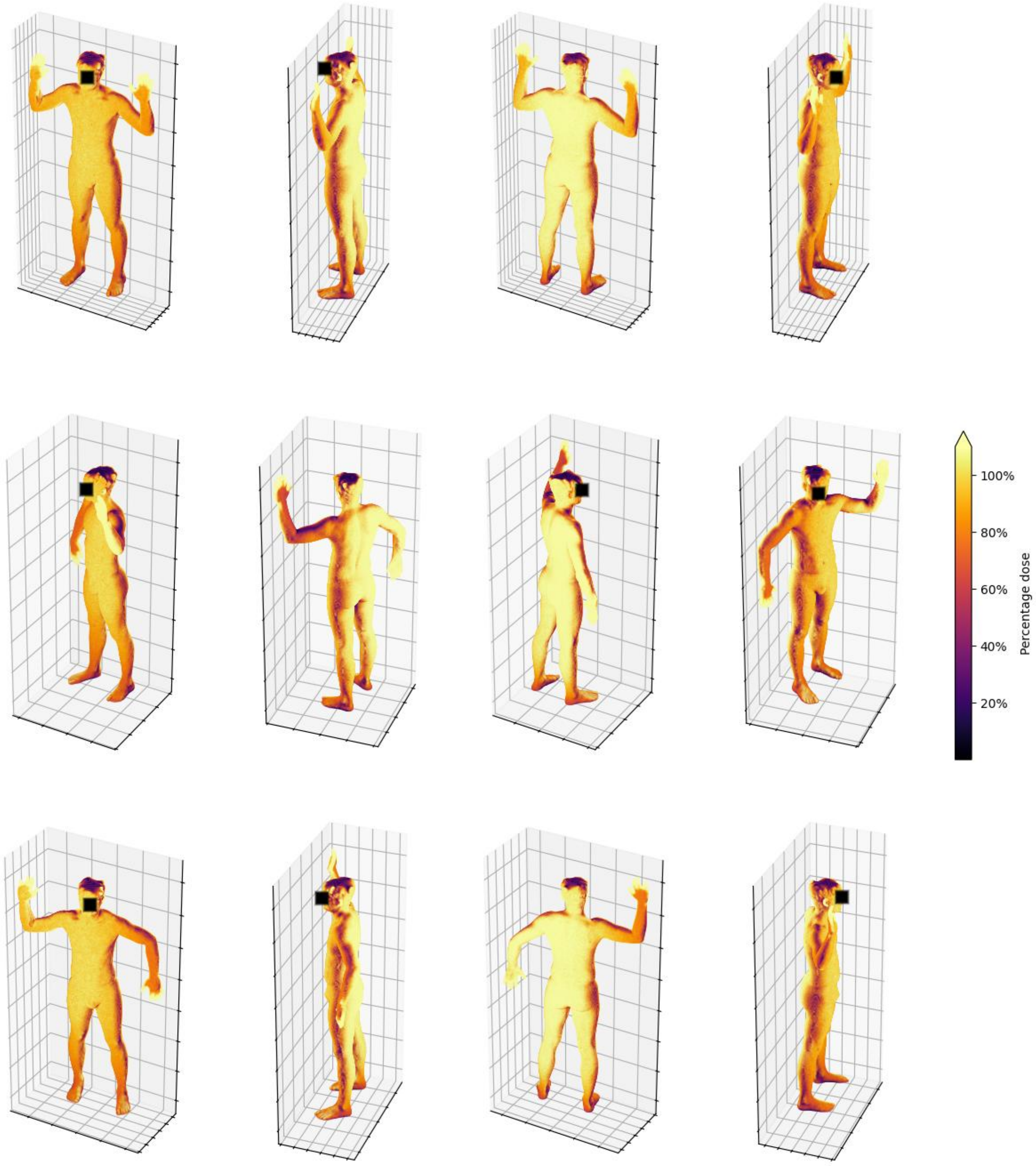

Supporting Materials Figure 1. Example paired dual-field dose distributions for participant 1, in AP/PA position (top), RAO/LPO position (middle) and RPO/LAO position (bottom). Facial features have been hidden to preserve participant anonymity.

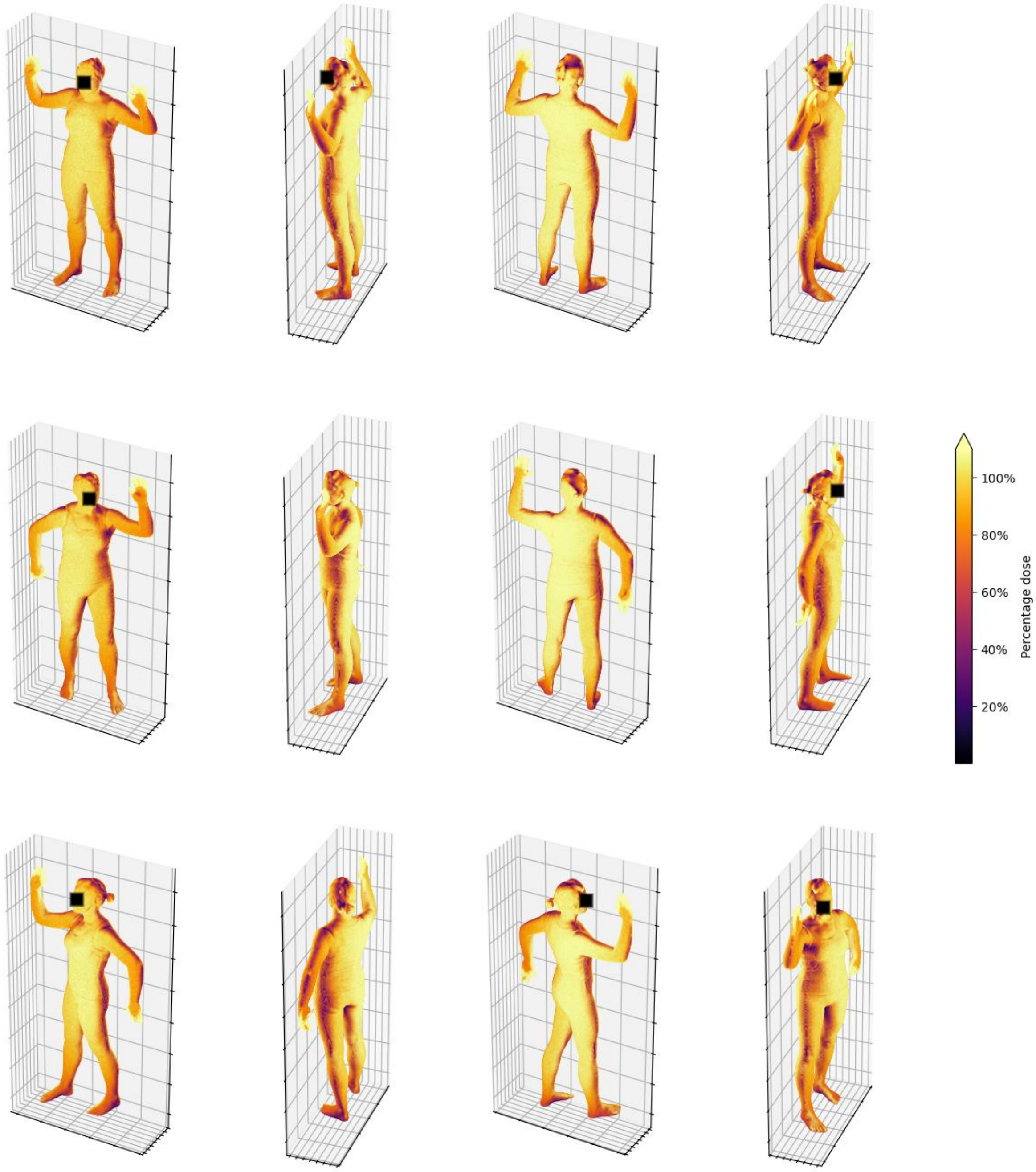

Supporting Materials Figure 2 Example paired dual-field dose distributions for participant 2, in AP/PA position (top), RAO/LPO position (middle) and RPO/LAO position (bottom). Facial features have been hidden to preserve participant anonymity.

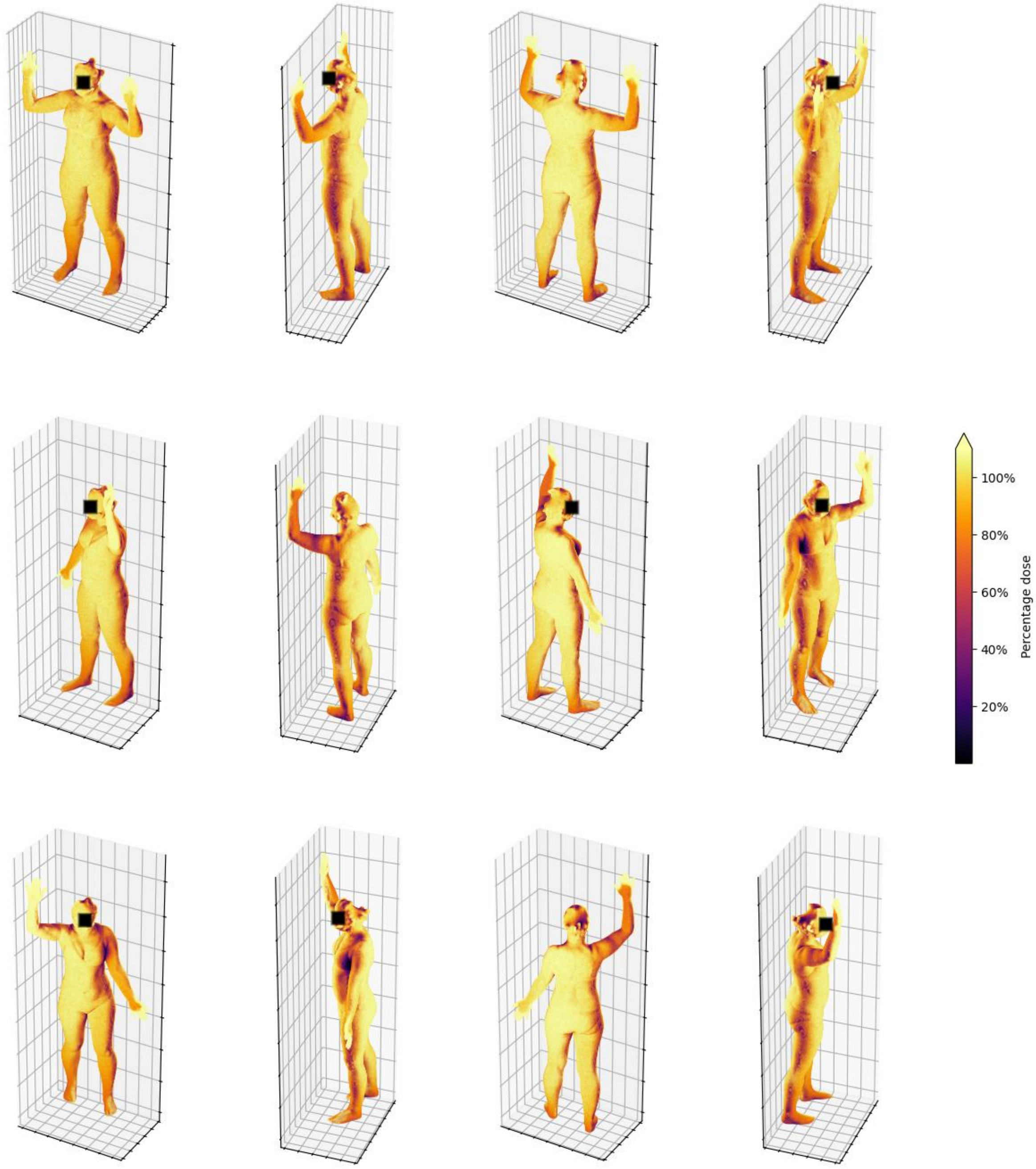

Supporting Materials Figure 3. Example paired dual-field dose distributions for participant 3, in AP/PA position (top), RAO/LPO position (middle) and RPO/LAO position (bottom). Facial features have been hidden to preserve participant anonymity.

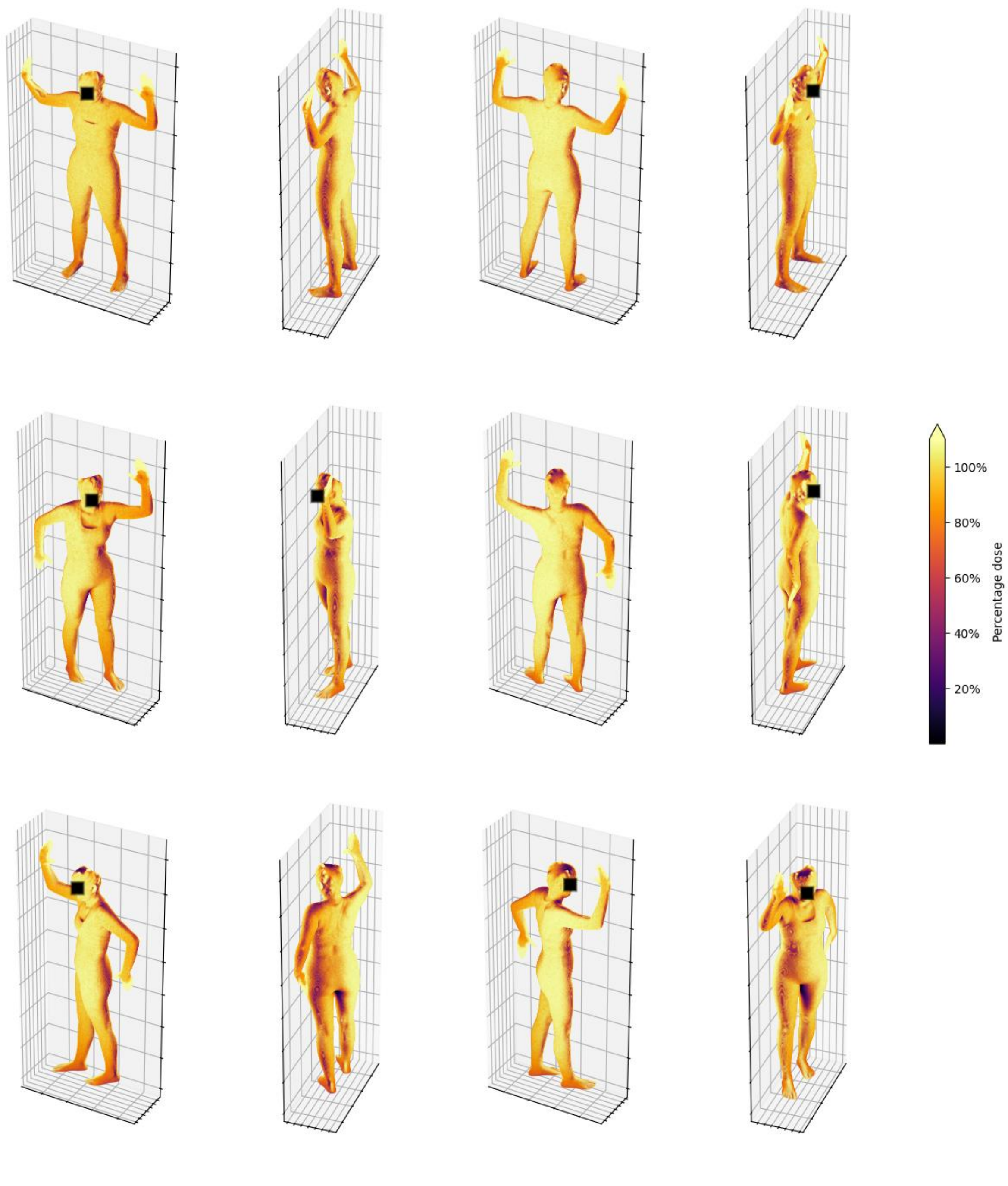

Supporting Materials Figure 4 Example paired dual-field dose distributions for participant 5, in AP/PA position (top), RAO/LPO position (middle) and RPO/LAO position (bottom). Facial features have been hidden to preserve participant anonymity.

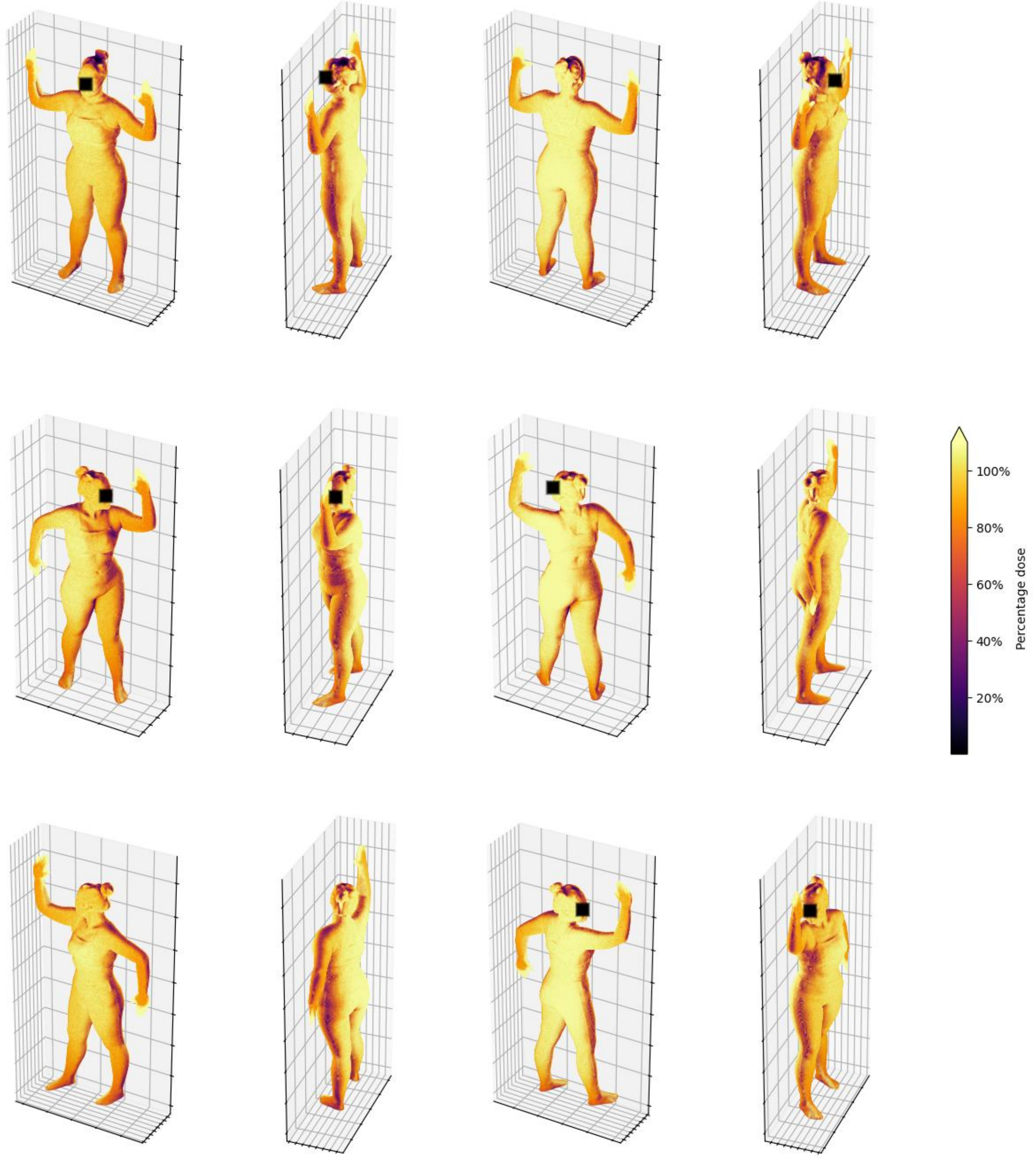

Supporting Materials Figure 5 Example paired dual-field dose distributions for participant 6, in AP/PA position (top), RAO/LPO position (middle) and RPO/LAO position (bottom). Facial features have been hidden to preserve participant anonymity.

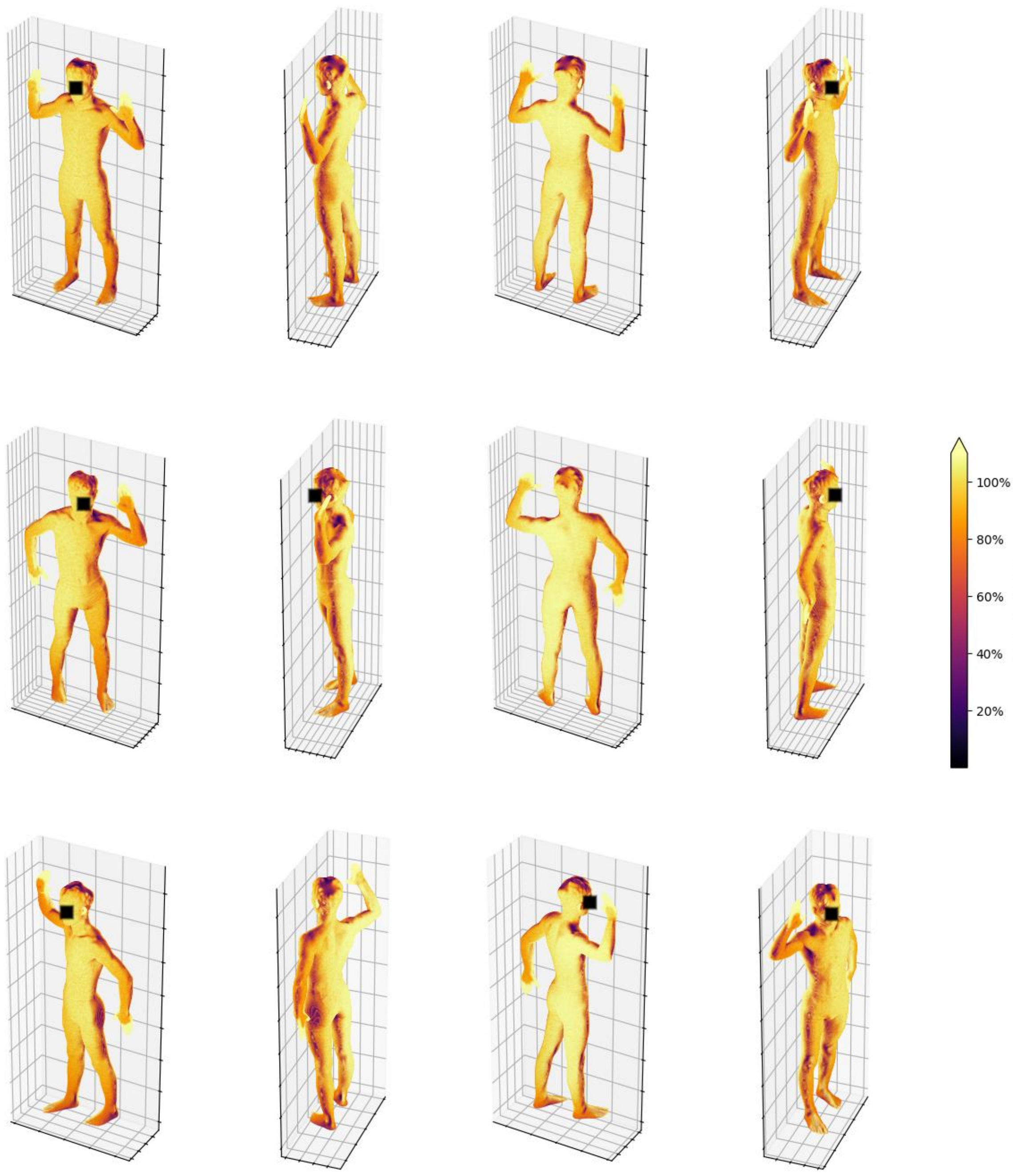

Supporting Materials Figure 6 Example paired dual-field dose distributions for participant 7, in AP/PA position (top), RAO/LPO position (middle) and RPO/LAO position (bottom). Facial features have been hidden to preserve participant anonymity.

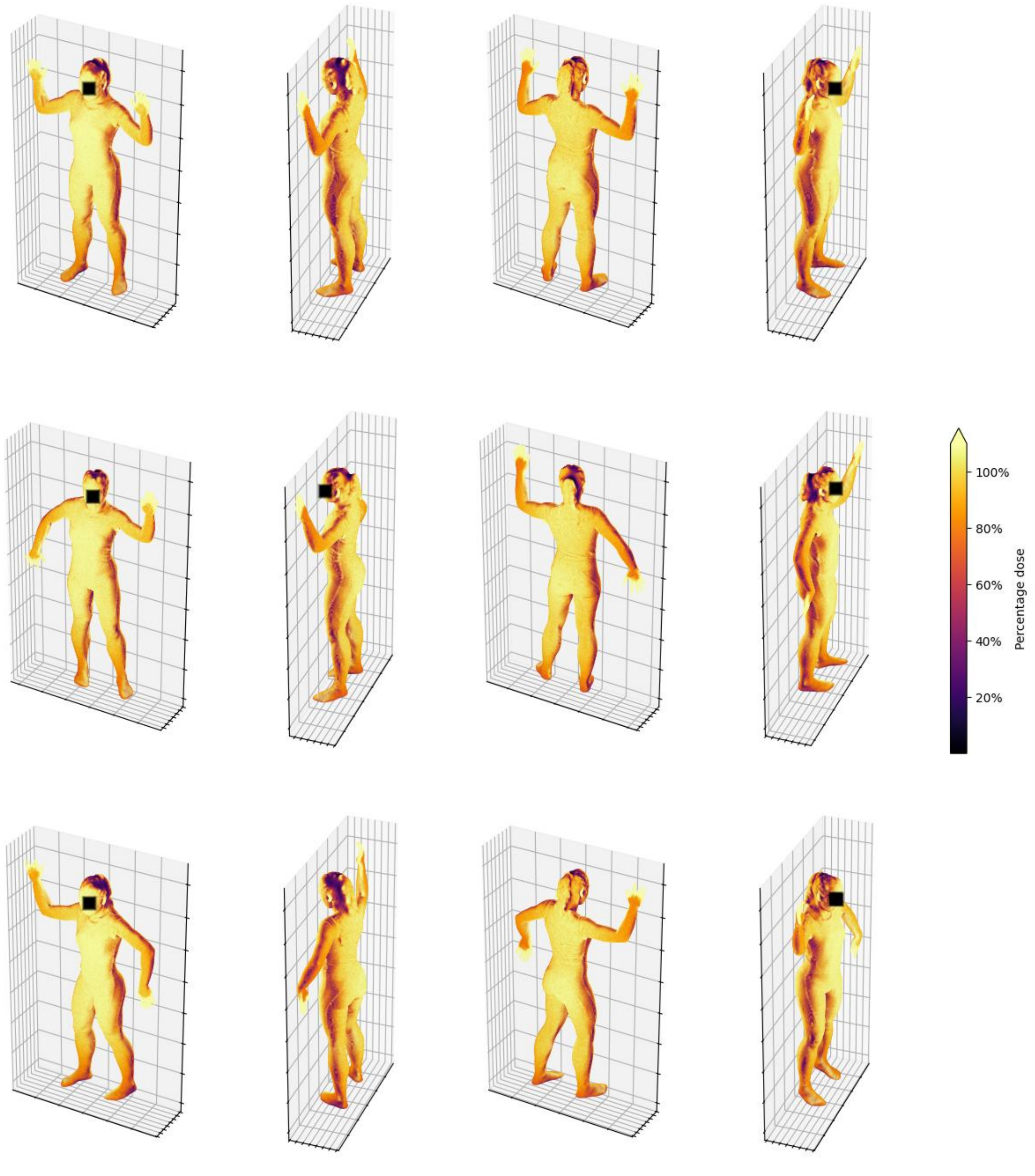

Supporting Materials Figure 7 Example paired dual-field dose distributions for participant 8, in AP/PA position (top), RAO/LPO position (middle) and RPO/LAO position (bottom). Facial features have been hidden to preserve participant anonymity.
